# Supplementary material for: Assessing the school food environment and its role on healthy eating behaviours among school age children in Dar es Salaam, Tanzania
Source: PLoS One. 2025 Jul 9;20(7):e0321702. doi: 10.1371/journal.pone.0321702 (PMC12240332; doi:10.1371/journal.pone.0321702)
Supplement: Attachment — Submitted filename: Response Matrix Sheet_16_05_2025.docx [file pone.0321702.s002.docx]

**Response Matrix Sheet**

| **S/N** | **Comments** | **Response** |
| --- | --- | --- |
| 1 | We note that the grant information you provided in the ‘Funding Information’ and ‘Financial Disclosure’ sections do not match. | Correction was made to match funding information. The study received grants from the Vanke School of Public Health at Tsinghua University. |
| 2 | Thank you for stating the following financial disclosure: “We acknowledge funding from Bright future “ Please state what role the funders took in the study. If the funders had no role, please state: "The funders had no role in study design, data collection and analysis, decision to publish, or preparation of the manuscript." | Funder had a role in reviewing the manuscript. |
| 3 | In the online submission form, you indicated that “Data cannot be shared publicly, is available upon request” All PLOS journals now require all data underlying the findings described in their manuscript to be freely available to other researchers, either 1. In a public repository, 2. Within the manuscript itself, or 3. Uploaded as supplementary information. This policy applies to all data except where public deposition would breach compliance with the protocol approved by your research ethics board. If your data cannot be made publicly available for ethical or legal reasons (e.g., public availability would compromise patient privacy), please explain your reasons on resubmission and your exemption request will be escalated for approval. | This is well noted, data is now available and shared publicly at Tanzania Food and Nutrition Centre repository as well as in the manuscript itself. Find the link for accessing Tanzania Food and Nutrition Centre repository <https://www.tfnc.go.tz/publications/20> |
| 4 | When completing the data availability statement of the submission form, you indicated that you will make your data available on acceptance. We strongly recommend all authors decide on a data sharing plan before acceptance, as the process can be lengthy and hold up publication timelines. Please note that, though access restrictions are acceptable now, your entire data will need to be made freely accessible if your manuscript is accepted for publication. This policy applies to all data except where public deposition would breach compliance with the protocol approved by your research ethics board. If you are unable to adhere to our open data policy, please kindly revise your statement to explain your reasoning and we will seek the editor's input on an exemption. Please be assured that, once you have provided your new statement, the assessment of your exemption will not hold up the peer review process. | All authors agreed data to be available publicly at Tanzania Food and Nutrition Centre |
| 5 | Please ensure that you refer to Figure 2 in your text as, if accepted, production will need this reference to link the reader to the figure. | Figure 2 is referred in the text, see line 415 |
| 6 | Please ensure that you refer to Table 1 in your text as, if accepted, production will need this reference to link the reader to the Table. | Table 1 is referred in the text, see line 258 |
